# Preparation and Characterization of Chitosan-Modified Bentonite Hydrogels and Application for Tetracycline Adsorption from Aqueous Solution

**DOI:** 10.3390/gels10080503

**Published:** 2024-07-28

**Authors:** Xuebai Guo, Zhenjun Wu, Zheng Lu, Zelong Wang, Shunyi Li, Freeman Madhau, Ting Guo, Rongqican Huo

**Affiliations:** 1 Henan Vocational College of Water Conservancy and Environment, Zhengzhou 450008, China; guoxuebai@126.com; 2 School of Environmental Engineering, Henan University of Technology, Zhengzhou 450001, China; luzheng1042@163.com (Z.L.); jordanfm2015@gmail.com (F.M.); 17513282098@163.com (T.G.); 15935196916@163.com (R.H.); 3 Post-Doctoral Workstation, Henan Xinanli Security Technology Co., Ltd., Zhengzhou 450001, China; 4 School of Ecology and Environment, Zhengzhou University, Zhengzhou 450001, China; wzl765966763@gmail.com (Z.W.); lsy76@zzu.edu.cn (S.L.)

**Keywords:** bentonite, chitosan, adsorption, tetracycline, characterization

## Abstract

The “sol–gel method” was used to prepare spherical chitosan-modified bentonite (SCB) hydrogels in this study. The SCB hydrogels were characterized and used as sorbents to remove tetracycline (TC) from aqueous solutions. The adsorbents were characterized by SEM, XRD, FTIR, TG, and BET techniques. Various characterization results showed that the SCB adsorbent had fewer surface pores and a specific surface area that was 96.6% lower than the powder, but the layered mesoporous structure of bentonite remained unchanged. The adsorption process fit to both the Freundlich model and the pseudo-second-order kinetic model showed that it was a non-monolayer chemical adsorption process affected by intra-particle diffusion. The maximum monolayer adsorption capacity determined by the Langmuir model was 39.49 mg/g. Thermodynamic parameters indicated that adsorption was a spontaneous, endothermic, and entropy-increasing process. In addition, solid–liquid separation was easy with the SCB adsorbent, providing important reference information for the synthesis of SCB as a novel and promising adsorbent for the removal of antibiotics from wastewater at the industrial level.

## 1. Introduction

Recently, antibiotic removal technology has emerged as a hot research topic in wastewater treatment, attracting increasing global public attention. Because it is difficult to biodegrade antibiotics, they cannot be removed using traditional biotechnology methods [1]. As a result, antibiotic wastewater treatment is a major issue in water pollution treatment. Antibiotic wastewater endangers the health of aquatic organisms and has a negative impact on human health via the food chain [2]. As a result, antibiotic removal in wastewater has received a lot of attention. Antibiotics are classified into tetracyclines, sulfonamides, macrolides, quinolones, and beta-lactam antibiotics based on their chemical structure. Tetracycline antibiotics (tetracyclines, TCs) are the most commonly used among them, and include tetracycline (TC), chlortetracycline (CTC), oxytetracycline (OTC), and others. Except for its microbial metabolism, the residual tetracycline in the environment is the result of human production and life [3], such as pharmaceutical wastewater, medical wastewater, domestic sewage, livestock and poultry breeding wastewater, and aquaculture wastewater [4].

The residual TC in water can be extremely harmful. Many studies on TC control have been conducted in order to protect human health and the ecological environment. Adsorption [5], biodegradation [6], advanced oxidation [7], photodegradation [8], membrane separation [9], and other techniques are currently being used to remove antibiotics from wastewater. When comparing various treatment methods, the adsorption method has drawn attention because of its advantages, which include a high removal efficiency, ease of operation, low cost, and broad application prospects [10]. Additionally, the adsorbent can be recycled and regenerated, lowering the cost of wastewater treatment. At the moment, activated carbon [11], MOFs [12], bentonite [13], etc., are the most frequently utilized adsorbents for treating TC wastewater. Although activated carbon has an outstanding adsorption efficiency and is frequently used in wastewater treatment, its use is restricted by the fact that it has a relatively high production cost and is easily lost. A large specific surface area and high porosity are advantages of MOFs, but their preparation is labor-intensive and expensive to produce. Bentonite, a low-cost clay mineral with a large specific surface area, strong adsorption ability, and ion exchange capabilities, is an environmentally acceptable adsorbent [14].

In recent years, researchers have used bentonite’s natural adsorption properties to remove heavy metals, inorganic pollutants, organic pollutants, and nonferrous compounds from wastewater [15]. However, because bentonite is extremely hydrophilic, it easily combines with water molecules, resulting in a poor adsorption capacity for organic contaminants in wastewater [16]. Furthermore, natural bentonite powder is difficult to separate from liquid, limiting its application [17]. As a result, bentonite is frequently changed for practical applications in order to improve its organic pollutant adsorption ability. Its adsorption ability is greatly increased after modification, and it is widely employed in the treatment of diverse wastewaters.

Natural bentonite is particularly hydrophilic, allowing it to rapidly bond to the water molecules in a solution, which is detrimental to organic matter transport and the adsorption on its surface [18]. Organic modification can improve its lipophilic and hydrophobic properties, as well as its tetracycline adsorption ability [19]. However, the powdered modified clay will still form a suspension in the wastewater, making solid–liquid separation difficult, and it is difficult to avoid adsorbent loss during the recovery process, increasing the cost of adsorbent regeneration and making bentonite unsuitable for practical application. Certain methods for preparing powder-modified soil can efficiently overcome this problem. 

Chitosan is a natural polymer with excellent adsorption characteristics for heavy metals, organic contaminants, and so on [20]. It is used to modify bentonite, which can not only improve bentonite’s tetracycline adsorption ability, but also increase its hydrophobicity and minimize the difficulty of solid–liquid separation after adsorption, with promising application prospects [21]. Natural calcium-based bentonite was modified with Na_2_CO_3_ and chitosan, and powdered chitosan modified bentonite was produced. The hydrophobicity of powdered bentonite was increased through this modification; however, this could not totally solve the problems of solid–liquid separation and adsorbent loss during the recovery process. To further widen its applicability, the sol–gel process was used to synthesize spherical chitosan-modified bentonite hydrogels, which may solve the problems of separation and recovery [22,23].

The objective of this study was to investigate the sorption behavior of TC on SCB hydrogels. The results of this study are to provide a method to synthesize novel chitosan-bentonite hydrogels to solve the problem of the difficulty of separating traditional bentonite adsorbents from aqueous solutions. The adsorbent’s adsorption effectiveness in simulated tetracycline wastewater was investigated to give a theoretical basis for the treatment of actual tetracycline wastewater.

## 2. Results and Discussion

### 2.1. Sol–Gel Process Optimization

#### 2.1.1. Optimization of the Mass Ratio of Chitosan/Bentonite

In this experiment, the impact of various chitosan/bentonite mass ratios on the adsorption effect was investigated. The adsorption capacity of the spherical adsorbent gradually decreased as the mass ratio of chitosan/bentonite increased; it was 17.61 mg/g at the mass ratio of 1:50, the removal efficiency was 88.25%, and the remaining mass ratio significantly decreased because the combination of chitosan and bentonite occupied part of the active sites, causing decreases in the specific surface area and pore volume of the spherical adsorbent [24]. Taking into account the adsorption effect and preparation cost, the spherical adsorbent was produced by selecting a mass ratio of 1:50 of chitosan/bentonite as the optimum mass ratio.

#### 2.1.2. Comparison of Drying Methods

In this study, the effects of oven-drying and freeze-drying on the adsorption effect of the spherical adsorbent were investigated, and the results are shown below.

**Figure 1:**
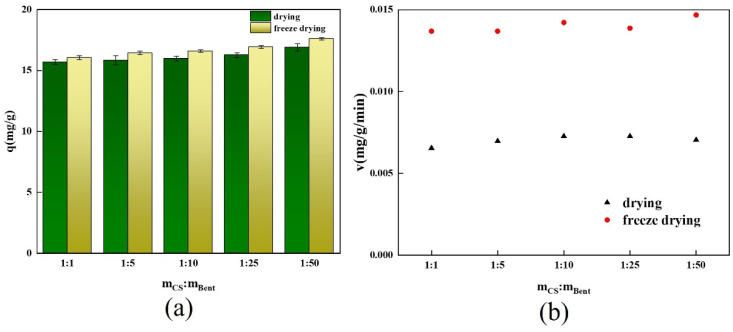
(**a**) Effects of different drying methods of spherical chitosan-modified bentonite and (**b**) comparison of adsorption rates of different drying methods.

Figure 1a compares the equilibrium adsorption capacities of the adsorbent obtained by drying and freeze-drying after equilibrium adsorption was reached. The adsorption capacities of the spherical adsorbents obtained by freeze-drying with different chitosan/bentonite mass ratios were slightly higher than the adsorbents obtained by drying.

Figure 1b illustrates a comparison of the adsorption rates of the two drying processes’ spherical adsorbents. According to the findings, the adsorption rate of the freeze-dried adsorbents was approximately twice that of the dried adsorbents. Because of the high temperature used for drying the gel adsorbents, the internal water evaporation of the adsorbents was excessively rapid, resulting in a partial collapse of the internal structure of the calcium alginate gel adsorbents and the destruction of part of the pore structure. As a result, the spherical adsorbent’s mass transfer resistance increased, resulting in a reduced adsorption rate [25]. In contrast, the freeze-drying process froze the gel spheres after cross-linking, maintaining the internal water and particle structure. The water in the gel spheres was dispersed more slowly by sublimation during the vacuum freeze-drying process, preventing the internal structure of the spherical adsorbent from collapsing [4]. As a result, the freeze-drying method was chosen as the drying method for the spherical adsorbents in this experiment due to its high adsorption capacity and quick adsorption equilibrium time.

#### 2.1.3. Optimization of Sodium Alginate Concentration

The effect of the sodium alginate concentration (*w*/*v*) on the adsorption capacity of the adsorbent was investigated in this experiment. The sodium alginate concentrations were set at 0.5%, 1%, 1.5%, 2%, and 2.5%, respectively. The optimum sodium alginate concentration was then determined by observing the morphological characteristics of the prepared gel microspheres. Figure S1 shows images of the gel microspheres made with various concentrations of sodium alginate.

The particle size of the gel micro-spheres gradually increased as the concentration of sodium alginate increased, as did the mechanical strength of the gel micro-spheres. When the sodium alginate content was 0.5%, the mechanical strength of the resulting gel microspheres was insufficient and readily shattered. When the sodium alginate concentration reached 1%, the gel microspheres and freeze-dried spherical adsorbents retained their morphology and were not easily disrupted [26]. When the sodium alginate content was raised to more than 2%, the viscosity of the solution rose, causing issues with dripping and considerable variances in the sizes of the gel spheres [27]. When the sodium alginate concentration exceeded 2.5%, the solution had to be added while still hot, and the viscosity of the solution was too high to produce spherical adsorbents.

Figure S2 shows the results of the tetracycline adsorption experiments using spherical adsorbents made from various amounts of sodium alginate. When the concentration of sodium alginate was less than 1%, the equilibrium adsorption capacity and removal efficiency varied less, and as the concentration of sodium alginate grew, the adsorbent’s adsorption capacity gradually decreased. The quantity of sodium alginate bound to the chitosan modified bentonite rose as the concentration of sodium alginate increased, occupying part of the adsorption site [28]. The carboxyl groups in sodium alginate’s structure also quickly cross-linked with the calcium chloride solution to create a denser mesh structure due to the high sodium alginate concentration, which raised the solid–liquid phase mass transfer barrier and reduced the adsorption effect [29]. In conclusion, sodium alginate is expensive and the generated adsorbents were prone to rupture during the adsorption process, which do not meet the conditions of use.

#### 2.1.4. Optimization of Calcium Chloride Solution Concentration

In this experiment, the impact of the CaCl_2_ solution concentration (*w*/*v*) on the adsorption effectiveness was examined. The concentration of the CaCl_2_ solution was tested at 1%, 2%, 3%, 4%, and 5%, respectively. The calcium chloride concentration had a negligible impact on the effectiveness of tetracycline removal when the CaCl_2_ concentration was less than 2%. The spherical adsorbent’s adsorption effectiveness started to decline as the CaCl_2_ concentration gradually rose. This was because the amount of cross-linking within the gel spheres steadily increased as the CaCl_2_ concentration rose. The adsorbent mesh structure became denser as the stretching degree increased, increasing the solid–liquid phase mass transfer resistance. This prevented tetracycline molecules from entering the particle and interacting with the chitosan-modified bentonite, resulting in a decrease in the adsorption effect [29]. After considering the adsorption effect and the mechanical strength of the adsorbent, a 2% (*w*/*v*) CaCl_2_ solution was used to prepare the spherical adsorbent.

#### 2.1.5. Adsorption Capacity of Different Forms of Adsorbents

Cross-linking helps to prepare bentonite into spherical adsorbent, but it decreases the adsorption properties of bentonite. In order to investigate the effect of cross-linking on the adsorption properties of bentonite, the adsorption properties of powder adsorbents and spherical adsorbents of bentonite were compared in experiments. As shown in Table 1, when the dosage of both adsorbents was 5 g/L and the initial concentration of tetracycline was 100 mg/L and below, the adsorption amount (qe) and removal efficiency of the two adsorbents on tetracycline were similar. The equilibrium adsorption amount (qe) of the spherical adsorbent was 79.6% of that of the powder adsorbent at an initial concentration of 150 mg/L of tetracycline. The increase in the equilibrium adsorption capacity (q_e_) of the spherical adsorbent decreased with a further increase in the initial concentration of tetracycline (C_0_), while the q_e_ of the powder adsorbent maintained a high increase. When the initial concentration of tetracycline reached 600 mg/L, the q_e_ of the spherical adsorbent was only 37.4% of that of the powder adsorbent. This shows that the spherical adsorbent was effective in treating wastewater with a tetracycline concentration of less than 150 mg/L. The concentrations of tetracycline in actual wastewater from livestock and poultry farming, aquaculture, and pharmaceuticals are much less than 150 mg/L. Therefore, the spherical adsorbent has a broad potential for engineering applications because of its convenient solid–liquid separation and its advantage of no adsorbent loss.

The maximum adsorption capacity of the spherical bentonite-chitosan adsorbent prepared in this study for tetracycline was about 100 mg/g. It has been shown that the powder bentonite adsorbent has a higher adsorption capacity for tetracycline of up to 476 mg/g [14]. The equilibrium adsorption capacity of the spherical bentonite adsorbent for tetracycline in the present study was low, which was mainly due to the low pore volume of only 0.008 cm^3^/g of the spherical adsorbent. In the process of preparing the spherical adsorbent, the encapsulation of calcium alginate resulted in the clogging of most of the small pores of the spherical adsorbent, and a decrease in the pore volume led to a decrease in the physical adsorption capacity of adsorbent, which was an important reason for the decrease in the adsorption capacity. However, after the treatment of tetracycline wastewater, the spherical adsorbent could be separated from water by gravitational settling, reducing the loss of the adsorbent. Compared with the powder adsorbent with a larger adsorption capacity, the spherical adsorbent has a higher engineering application value.

### 2.2. Characterization of Results Analysis

**Figure 2:**
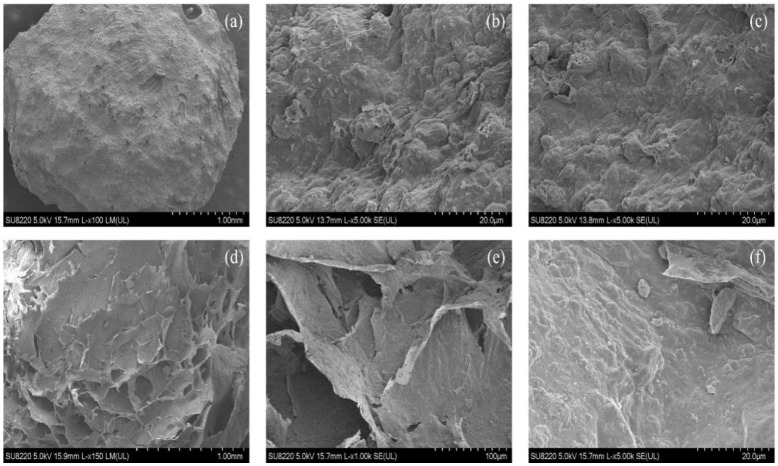
SEM images of spherical chitosan-modified bentonite surfaces (**a**–**c**) and cross-sections (**d**–**f**).

Figure 2a–c show that the spherical chitosan-modified bentonite had an uneven surface, which can provide specific adsorption sites for pollutants. Pore channels, which are routes for water molecules to enter and exit, also existed on the surfaces of the adsorbents. The adsorbents had a reticulated structure within, as shown in Figure 2d–f, which effectively immobilized the chitosan-modified bentonite powder [4]. The irregularly shaped bentonite powder was put onto the calcium alginate reticulation structure under magnification.

**Figure 3:**
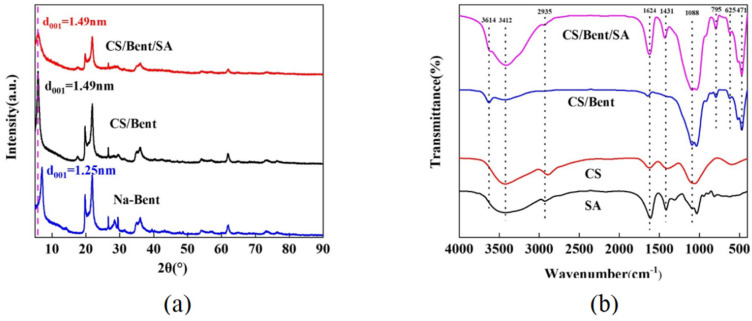
(**a**) XRD of sodium−modified bentonite, chitosan−modified bentonite (1:50) and spherical chitosan−modified bentonite; (**b**) FT−IR diagram of sodium alginate, chitosan, chitosan−modified bentonite, and spherical chitosan−modified bentonite.

The XRD spectra of the sodium-modified bentonite, chitosan-modified bentonite (1:50), and spherical chitosan-modified bentonite had practically the same peak shapes and did not differ considerably, as shown in Figure 3a. The findings of the investigation showed that the chitosan modification and cross-linking process of sodium alginate with CaCl_2_ did not disrupt the bentonite’s inter-layer skeletal structure, but the position of the d001 crystalline surface characteristic diffraction peak shifted [25]. The 2θ angles of the crystalline diffraction peaks of the sodium-modified bentonite, chitosan-modified bentonite, and spherical chitosan-modified bentonite d001 were 7.08°, 5.94°, and 5.96°, respectively. According to Bragg’s equation, the layer spacings of the sodium-modified bentonite, chitosan-modified bentonite, and spherical chitosan-modified bentonite were 1.25 nm, 1.49 nm, and 1.49 nm, respectively. The layer spacing of the sodium-modified bentonite modified by chitosan increased dramatically, indicating that the chitosan penetrated the sodium-modified bentonite inter-layer and propped it up, and that the chitosan modification was successful [1]. The layer spacing of the spherical adsorbent, on the other hand, did not vary much, indicating that the sodium alginate did not enter the bentonite inter-layer.

Figure 3b shows FT-IR graphs of the sodium alginate, chitosan, chitosan-modified bentonite, and spherical chitosan-modified bentonite. Among them, the peak at 3614 cm^−1^ is the stretching vibration peak of Al-O-H, the peak at 3412 cm^−1^ is the superposition of the stretching vibration peaks of -OH and -NH_2_, and the peak at 2935 cm^−1^ is the superposition of the asymmetric stretching vibrations of -CH_3_ and -CH_2_ in the chitosan molecule and the aliphatic C-H stretching group in the sodium alginate molecule. The peaks at 1624 cm^−1^ and 1431 cm^−1^ correspond to the asymmetric and symmetric vibrations of the carboxyl group in the sodium alginate molecule, respectively, which are the characteristic peaks of the sodium alginate molecule appearing on the spherical adsorbent. The peak at 471 cm^−1^ is the vibration of the Si-O tetrahedron in the Si-O-Si structure, the peak at 625 cm^−1^ is the bending vibration peak of Si-O-Al, the peak at 795 cm^−1^ corresponds to the symmetric vibration of Si-O, and the peak at 1088 cm^−1^ is the peak of the stretching vibration of Si-O. The peak shapes of the chitosan-modified bentonite and spherical chitosan-modified bentonite were generally consistent from 1088 to 471 cm^−1^, indicating that the cross-linking of sodium alginate with CaCl_2_ for granulation did not change the skeletal structure of the silica–oxygen tetrahedral and aluminums–oxygen octahedral of bentonite [30]. In summary, the FT-IR results indicate that the sol–gel granulation process did not change the basic structure of the chitosan-modified bentonite.

**Figure 4:**
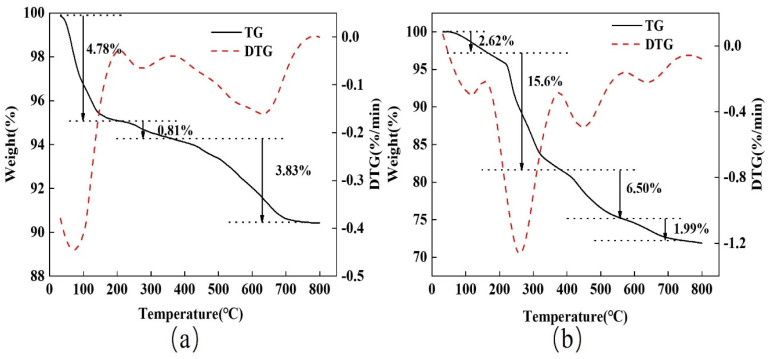
TG−DTG diagram of chitosan-modified bentonite (1:50) (**a**) and spherical chitosan-modified bentonite (**b**).

The TG-DTG plots for the chitosan-modified bentonite (1:50) and spherical chitosan-modified bentonite are shown in Figure 4. Figure 4a depicts three mass loss processes in the chitosan-treated bentonite. The temperature range from 30 °C to 200 °C correlates to the DTG curve’s first major heat absorption peak with a mass loss of 4.78%, which corresponds to the DTG curve’s first strong heat absorption peak, which is related to the loss of adsorbed water from the chitosan-modified bentonite. The temperature range from 200 °C to 360 °C has a weaker heat absorption peak, with a mass loss of 0.81% due to the decomposition of some of the loaded chitosan into CO_2_, CO, and H_2_O. The temperature range 360−780 °C has a broad heat absorption peak, with a mass loss of 3.83% caused by the decomposition of the remaining chitosan and the loss of water from the bentonite structure. Dehydroxylation caused the internal structure of bentonite to collapse, destroying its adsorption properties [27].

There were four mass loss processes in the spherical chitosan-modified bentonite, as shown in Figure 4b. There is a first heat absorption peak with a mass loss of 2.62% in the range from 30 to 160 °C, which is related to the loss of the spherical adsorbent’s adsorbed water. The decomposition of sodium alginate and chitosan causes a strong heat absorption peak in the range from 160 to 380 °C with a mass loss of 15.6%. There is a third heat absorption peak with a mass loss of 6.50% from 380 to 570 °C, which is associated with the loss of water from the adsorbent’s inter-layer and the decomposition of sodium alginate and chitosan. A weak heat absorption peak from 570 to 760 °C is associated with water loss from the bentonite structure. According to the TG curve, the spherical adsorbent lost significantly more weight than the powder, indicating that it contained some alginate, but its main component was still bentonite [31].

The specific surface area and pore volume significantly decreased following shaping, as shown in Table 2, while the pore size increased, possibly as a result of the calcium alginate blocking some of the smaller pores during the shaping process. The decreases in the specific surface area and pore volume caused a reduction in the physical adsorption of the granular adsorbent. [30].

### 2.3. Adsorption Experiment Results Analysis

#### 2.3.1. Effect of Different Dosage

**Figure 5:**
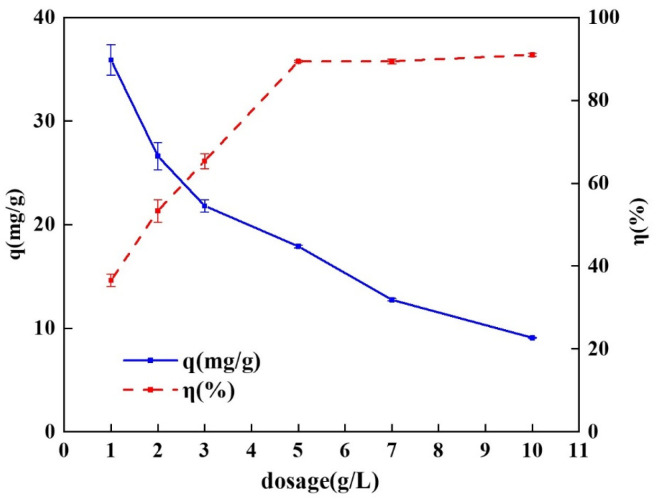
Effect of different dosage on adsorption of on adsorption of tetracycline by spherical chitosan-modified bentonite.

This experiment investigated the effects of different adsorbent dosing levels on the adsorption of tetracycline by the spherical chitosan-modified bentonite. Six gradients of 1 g/L, 2 g/L, 3 g/L, 5 g/L, 7 g/L, and 10 g/L were set for the static adsorption of tetracycline, and the results are shown in Figure 5. The adsorption capacity of the spherical chitosan-modified bentonite for tetracycline gradually decreased as the amount of adsorbent added gradually increased, with the adsorption capacity gradually decreasing from 35.91 mg/g to 9.08 mg/g, and the amount of change in the adsorption capacity gradually decreasing. The removal efficiency of tetracycline gradually increased from 36.57% to 89.43% with an increase in the dosage, and the change in the removal rate tended to flatten out when the dosage was higher than 5 g/L. Further increases in dosage did not increase the removal efficiency significantly, so 5 g/L was selected as the optimum dosage. The adsorption capacity was 17.91 mg/g and the removal rate was 89.43% at this time.

As the initial concentrations of the tetracycline simulated wastewater used in the adsorption experiments were all 100 mg/L, there was an excess of tetracycline molecules in the solution at a low adsorbent dosing. The adsorption sites on the surface of the spherical chitosan-modified bentonite were rapidly occupied by the tetracycline molecules and reached adsorption saturation, so the adsorption capacity was high in this case. When the dosage was increased to a certain level, the amount of tetracycline that could be adsorbed at the adsorption site was greater than the total amount of tetracycline in the solution, resulting in a shortage of tetracycline in the solution, a gradual decrease in the adsorption capacity, and a gradual increase in the removal rate [32].

#### 2.3.2. Effect of pH on the Adsorption

**Figure 6:**
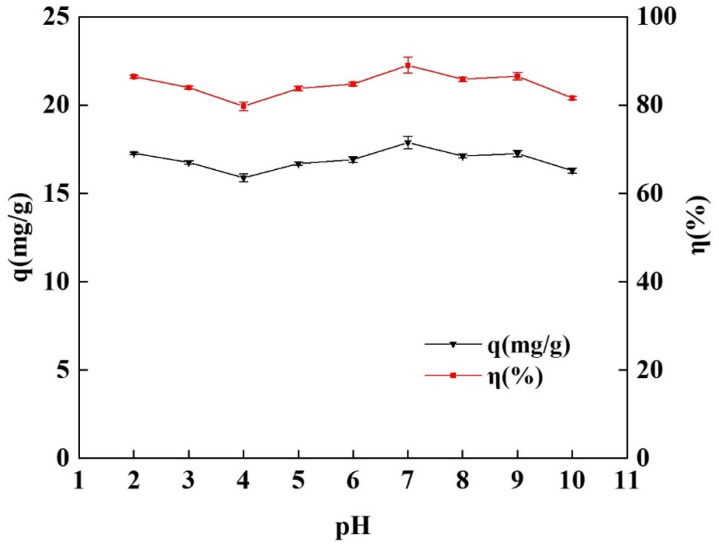
Effects of different initial pHs on adsorption of tetracycline by spherical chitosan-modified bentonite.

The effect of the initial pH of the tetracycline simulated wastewater on the performance of the spherical chitosan-modified bentonite in tetracycline adsorption was investigated in this experiment. The results of the static adsorption of tetracycline on nine gradients of initial pHs = 2–10 are shown in Figure 6. As the acidity of the solution decreased further, the adsorption capacity and removal efficiency of the spherical adsorbent for tetracycline gradually increased, peaking at 17.88 mg/g and 89.04%, respectively, at pH = 7. The reaction system became alkaline as the pH increased, and the adsorption capacity and removal efficiency gradually decreased. However, the overall removal efficiency remained greater than 80%, indicating that the spherical adsorbent had a wide pH adaptation range for tetracycline adsorption [11]. As a result, the subsequent experiments were conducted at an initial pH of 7.

#### 2.3.3. Effect of the Adsorption Times

**Figure 7:**
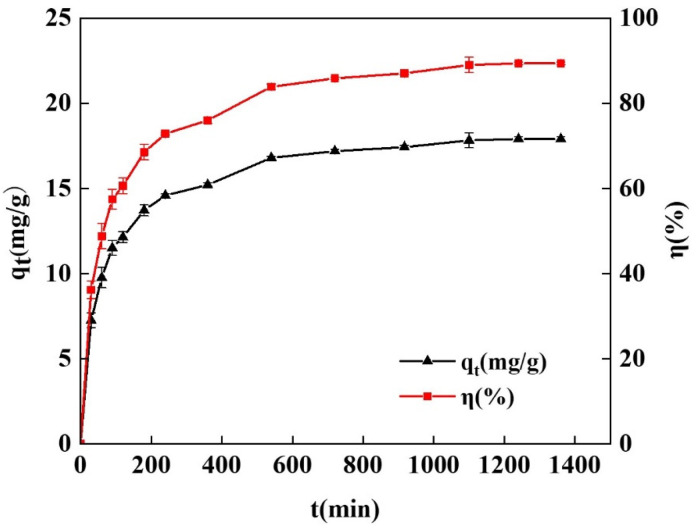
Effects of different adsorption times on adsorption of tetracycline by spherical chitosan-modified bentonite.

In this work, the efficacy of the spherical chitosan-modified bentonite in adsorbing tetracycline was examined in relation to various adsorption times. As shown in Figure 7, samples were taken routinely during the testing process, and the sampling interval was gradually increased as the adsorption duration increased until the concentration of tetracycline remained constant. In the first 240 min after the start of adsorption, the spherical adsorbent’s tetracycline adsorption capacity and removal efficiency climbed rapidly to 14.59 mg/g and 72.87%, and after 240 min, the growth rate of the adsorption capacity and removal efficiency slowed down. When the adsorption duration was raised to 1360 min as opposed to 1240 min, there was no discernible difference in the adsorption capacity and removal efficiency. Instead, the adsorption capacity and removal efficiency improved by 3.32 mg/g and 16.56%, respectively, during this 1000 min.

This was because the adsorbent initially had more blank adsorption sites, which could immediately bind to the tetracycline molecules in the simulated wastewater. As a result, the adsorption capacity and removal rate increased quickly. The number of adsorption sites in the blank rapidly reduced with an increasing adsorption time, as did the ability to bind to the tetracycline molecule, but the adsorption capacity and clearance rate gradually increased. At 1240 min, the tetracycline molecules finally completely occupied the blank adsorption sites within the adsorbent, attaining adsorption equilibrium with almost no change in adsorption capacity or removal rate [32].

### 2.4. Kinetics, Thermodynamic Analysis

#### 2.4.1. Adsorption Kinetics

**Figure 8:**
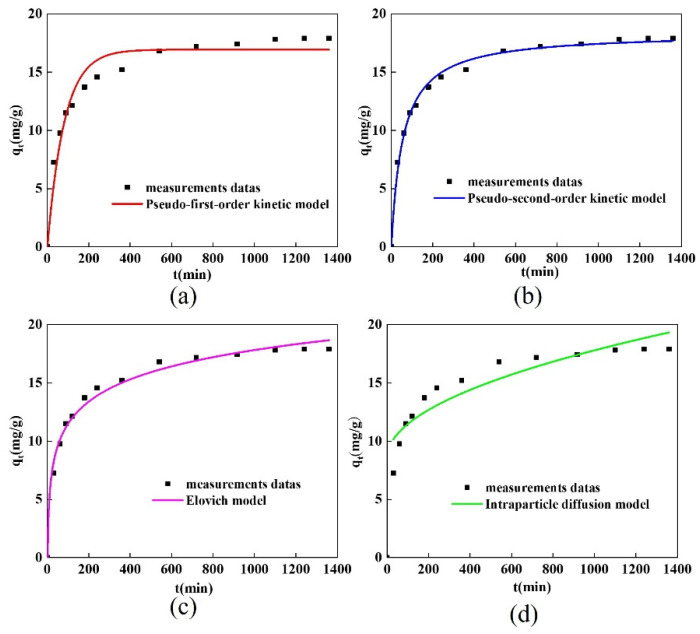
(**a**) Pseudo-first-order kinetic model, (**b**) pseudo-second-order kinetic model, (**c**) Elovich model, and (**d**) intraparticle diffusion model.

The pseudo-first-order kinetic model, pseudo-second-order kinetic model, Elovich model, and intra-particle diffusion model were used in the kinetic simulations of the tetracycline adsorption by the spherical chitosan-modified bentonite. Figure 8 displays the simulation’s results, while Table 3 displays the pertinent parameters.

Table 3 shows that the correlation coefficients for the pseudo-first-order kinetic model and the pseudo-second-order kinetic model were 0.952 and 0.994, respectively. The reaction process was a chemisorption process with an adsorption rate constant of 9.85 × 10^−4^ g·mg^−1^·min^−1^, according to the spherical chitosan-modified bentonite adsorption process on tetracycline, which was more consistent with the pseudo-second-order kinetic model. The Elovich model’s correlation coefficient was 0.989, and the model’s good fit suggested that the reaction process was a non-homogeneous diffusion process.

Intraparticle diffusion was not the only rate-limiting step in the adsorption process, as seen in Figure 8d, where the curve derived from the intraparticle diffusion model fit does not pass through the origin of the coordinates [1]. The correlation coefficient for the spherical adsorbent intraparticle diffusion model was higher than that for the chitosan-modified bentonite powder, however, at 0.846, indicating that the spherical adsorbent’s adsorption rate was more affected by intraparticle diffusion than the latter [31].

#### 2.4.2. Adsorption Isotherm

In this experiment, eight concentration gradients from 50 mg/L to 600 mg/L were set for each temperature for tetracycline static adsorption studies. Three temperature gradients of 20 °C, 30 °C, and 40 °C (293 K, 303 K, and 313 K) were also set. Table 4 below displays the results.

As can be observed in Table 4, both q_e_ and C_e_ steadily grew at the same temperature as the original concentration did. The adsorption sites on the surface of the adsorbent were, however, approaching saturation at this point, as seen by the progressive drop in qe and gradual increase in C_e_. The progressive rise in q_e_ with temperature, at the same initial concentration, suggested that the adsorption process benefited from the temperature increase.

**Figure 9:**
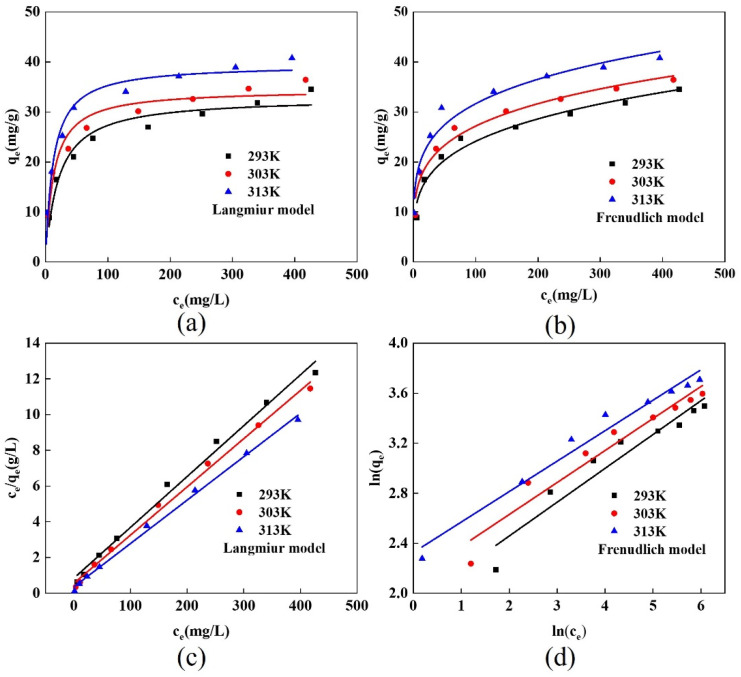
(**a**) Langmuir model, (**b**) Freundlich model, (**c**) linear fitting of Langmuir model, and (**d**) linear fitting of Freundlich model.

The adsorption process was simulated using the Freundlich and Langmuir models. Table 5 and Figure 9 display the pertinent variables and results. The Freundlich model had higher correlation coefficients than the Langmuir model at various temperatures. The Freundlich model’s 1/n values at various temperatures were all less than 0.5, indicating that the adsorption process could be carried out very simply.

#### 2.4.3. Adsorption Thermodynamics

Thermodynamic equations were used to calculate the thermodynamic parameters for the adsorption of tetracycline from the particulate adsorbent. Firstly, by plotting qe against ln(q_e_/C_e_), the *y*-axis intercept obtained from the fit was the thermodynamic equilibrium constant K_d_ at different temperatures. The results of the fit are shown in Figure S4a. The linear equation was obtained by linear fitting as y = −0.128 − x + 1.82 at 293 K, y = −0.138 − x + 2.61 at 303 K, and y = −0.138 – x + 3.33 at 313 K. K_d_ was 1.82, 2.61, and 3.33 at 293K, 303K, and 313K respectively, and the standard free energy (ΔG) was obtained by the equation: ΔG = −RT In K_L_ where R (8.314 J/mol.k) is the ideal gas constant; T (K) is the Kelvin temperature; and KL (L/mol) is the Langmuir constant. The standard enthalpy change (ΔH) and standard entropy change (ΔS) were further obtained by linearly fitting the Van’t Hoff equation with ln(K_d_) to 1/T, as shown in Figure S4b. The relevant thermodynamic parameters are shown in below.

The thermodynamic parameters for tetracycline adsorption from the particulate adsorbent were calculated using thermodynamic equations. The thermodynamic equilibrium constant K_d_ at various temperatures could first be determined by plotting qe against ln(q_e_/C_e_) and fitting the *y*-axis intercept. Figure S4 displays the fitting’s results (a). By fitting a straight line, the linear equation was found to be y = −0.128 − x + 1.82 at 293 K, y = −0.138 − x + 2.61 at 303 K, and y = −0.138 − x + 3.33 at 313 K. At 293 K, 303 K, and 313 K, respectively, K_d_ was 1.82, 2.61, and 3.33. The standard free energy (G) was calculated using the equation: G = −RT, where K_L_ (L/mol) is the Langmuir constant, T (K) is the Kelvin temperature, and R (8.314 J/mol.k) is the ideal gas constant. By linearly fitting the Van’t Hoff equation with ln(K_d_) to 1/T, the standard enthalpy change (H) and standard entropy change (S) were additionally obtained, as illustrated in Figure S4b. The following Table 6 displays the pertinent thermodynamic parameters.

The enthalpy change (ΔH) of the adsorption process, as determined by the pertinent thermodynamic parameters in Table 6, was G = 23.21 kJ/mol > 0, indicating that the reaction involving the spherical adsorption of tetracycline by a spherical adsorbent was a heat-absorption reaction process, and that raising the temperature was advantageous for the adsorption to proceed [33]. The adsorption process’ entropy change (ΔS) of S = 84.30 J/mol > 0 showed that it was an entropy-increasing process and that the adsorption system moved in the direction of escalating confusion [34]. Tetracycline adsorption by spherical adsorbents at various temperatures had a Gibbs free energy of less than 0, which suggests that the reaction can happen spontaneously [35]. In conclusion, the spontaneous heat-absorbing, entropy-increasing reaction mechanism that occurs during the adsorption of tetracycline onto spherical adsorbents is advantageous for the adsorption process.

## 3. Conclusions

In this research, spherical chitosan-modified bentonite hydrogels were successfully synthesized as adsorbents for TC adsorption. Our results revealed that chitosan-modified bentonite can form spherical adsorbents through the cross-linking of sodium alginate and calcium chloride, which improved the solid–liquid separation of the adsorbent from the solution. Moreover, the adsorption process was best described by the Freundlich isotherm model and the pseudo-second-order kinetic model. In conclusion, the results indicated that spherical chitosan-modified bentonite hydrogels may represent a novel strategy for removing tetracycline in wastewater.

## 4. Materials and Methods

### 4.1. Experimental Materials

The raw bentonite was obtained from Xinyang City, Henan province, China. The purification of bentonite was achieved using the gravity sedimentation method. Firstly, the raw bentonite was placed in an electric heating drying oven at 105 °C for 2 h. After cooling to room temperature, it was crushed with a ball mill to obtain bentonite ore powder. A sample of 100 g of the powder was mixed with deionized water to create a 10% bentonite slurry. This slurry was then vigorously stirred on a magnetic stirrer for 30 min to ensure complete dispersion. Following this, the mixture was allowed to stand for 30 min, after which, the upper liquid was separated using a centrifuge at 1500 rpm for 10 min. The upper clear liquid was discarded, and the lower solid was dried in a 105 °C oven for 24 h. After drying, it was crushed and sieved through a 200 mesh to obtain purified bentonite powder for future use.

Tetracycline (Ch.P), chitosan (≥95% deacetylated), acetic acid glacial (≥99.5%), sodium carbonate anhydrous (≥99.5%), and calcium chloride anhydrous (≥96%) were purchased from Shanghai Macklin Biochemical Technology Co., Ltd(Shanghai, China).

### 4.2. Preparation of Spherical Chitosan-Modified Bentonite

The sol–gel method was used in this study to synthesize spherical chitosan-modified bentonite with sodium alginate and calcium chloride as cross-linking agents. Firstly, five chitosan-modified bentonite powders with chitosan/bentonite mass ratios of 1:1, 1:5, 1:10, 1:25, and 1:50 were prepared. Secondly, 2.0 g of sodium alginate was weighed and added to 200 mL of deionized water and stirred vigorously at 60 °C for 2 h until the sodium alginate was completely dissolved, which was configured as a sodium alginate solution with a mass concentration of 1%. Then 10.0 g of chitosan-modified bentonite was added to the sodium alginate solution and stirred vigorously until the mixture was homogeneous. The mixed slurry of sodium alginate and chitosan-modified bentonite was squeezed and dripped into a certain mass concentration of calcium chloride solution using a syringe. The sodium alginate and calcium chloride rapidly underwent a cross-linking reaction to generate spherical adsorbents, and after 30 min of the cross-linking reaction, uniform spherical adsorbents were formed. Finally, the cross-linked adsorbents were rinsed repeatedly with deionized water to remove the remaining calcium chloride solution on the adsorbents’ surfaces, and the spherical chitosan-modified bentonite hydrogels were obtained after 24 h of drying.

### 4.3. Characterization of Absorbents

The surface morphologies of the sodium-modified bentonite and chitosan-modified bentonite were investigated using a Field Emission Scanning Electron Microscope (SU8200, Hitachi, Tokyo, Japan). The crystal structures of the adsorbents were characterized using X-ray diffractometer (D8 advance, Bruker, Berlin, Germany). The functional group structure information was analyzed by the potassium bromide press method using a Frontier FT-IR spectrometer (SPECTRUM 3, PerkinElmer, Waltham, MA, USA). Thermogravimetric analysis was carried out using a thermogravimetric analyzer (TGA-50H, Shimadzu, Kyoto, Japan). The specific surface area, pore volume, and pore size of the adsorbents were determined using a specific surface area and pore size tester (ASAP-2460, McMurray-Tick. Norcross, GW, USA).

### 4.4. Batch Adsorption Experiments

A stock solution of TC (100 mg/L) was prepared for the following batch experiments. Batch adsorption experiments for TC using SCB hydrogels were performed. TC solutions (50 mL and 100 mg/ L) with different adsorbent doses (1–10 g/L) were taken in conical flasks (250 mL) and stirred using a water bath shaker at 100 rpm at 30 ± 1 °C for 1240 min. The pHs of the solutions were adjusted in a range of 2–10 by using 0.1 mol/L HCl and 0.1 mol/L NaOH solutions. Kinetic studies of TC adsorption were performed by stirring 50 mL of TC solution (50–600 mg/L) in 250 mL conical flasks at 30 ± 1 °C for 1240 min until reaching equilibrium. The supernatants were measured with a liquid spectrometer at a wavelength of 357 nm (TC)

## Figures and Tables

**Table 1:** Ce and qe comparison under different initial concentrations.

C_0_ (mg/L)	Powder		Spherical	
	C_e_ (mg/L)	q_e_ (mg/g)	C_e_ (mg/L)	q_e_ (mg/g)
50	0.85	9.81	3.32	9.36
100	2.68	19.44	10.96	17.88
150	7.51	28.44	36.40	22.63
200	12.24	37.44	65.79	26.8
300	27.13	54.54	148.83	30.15
400	43.11	71.28	236.50	32.58
500	62.24	87.38	325.95	34.65
600	112.14	97.49	417.47	36.43

**Table 2:** Structural information of chitosan-modified bentonite (1:50) and spherical chitosan-modified bentonite.

Adsorbent	SBET (m^2^/g)	Pore Volume (cm^3^/g)	Pore Diameter (nm)
Chitosan-modified bentonite	73.23	0.216	11.79
Chitosan-modified bentonite adsorbents	2.50	0.008	12.94

**Table 3:** Parameters of adsorption kinetic model.

Model	Parameter		
Pseudo-first-order kinetic model	q_e_ (mg/g)	K_1_ (1/min)	R^2^
	16.94	0.012	0.952
Pseudo-second-order kinetic model	q_e_ (mg/g)	K_2_ (g·mg^−1^·min^−1^)	R^2^
	18.40	9.85·10^−4^	0.994
Elovich model	β (mg/g)	l n (αβ) (mg/g)	R^2^
	0.364	0.656	0.989
Intraparticle diffusion model	C (mg/g)	K_ip_ (m g·g^−1^·min^−0.5^)	R^2^
	8.55	0.292	0.846

**Table 4:** q_e_ and C_e_ at different temperatures and initial concentrations.

C_0_ (mg/L)	20 °C (293 K)		30 °C (303 K)		40 °C (313 K)	
	q_e_ (mg/g)	C_e_ (mg/L)	q_e_ (mg/g)	C_e_ (mg/L)	q_e_ (mg/g)	C_e_ (mg/L)
50	8.86	5.58	9.36	3.32	9.75	1.20
100	16.48	17.37	17.88	10.96	18.02	9.68
150	21.00	44.79	22.63	36.40	25.24	27.03
200	24.73	75.85	26.80	65.79	30.79	45.28
300	26.96	164.41	30.15	148.83	34.08	128.71
400	29.61	251.79	32.58	236.50	37.13	213.92
500	31.85	340.15	34.65	325.95	38.92	305.04
600	34.55	426.54	36.43	417.47	40.77	395.57

**Table 5:** Relevant parameters of Langmuir model and Freundlich model.

Temperature (K)	Langmiur Model			Freundlich Model		
	q_m_ (mg/g)	K_L_ (L/mg)	R^2^	K_F_(mg/g)	1/n	R^2^
293	32.88	0.049	0.941	7.77	0.246	0.961
303	34.55	0.078	0.931	9.86	0.220	0.962
313	39.49	0.084	0.925	12.18	0.207	0.957

**Table 6:** Thermodynamic parameters.

Temperature (K)	$∆G^{\theta }$ (kJ/mol)	$∆H^{\theta }$ (kJ/mol)	$∆S^{\theta }$
293	−1.46	23.21	84.30
303	−2.42		
313	−3.13		

## Data Availability

The raw data supporting the conclusions of this article will be made available by the authors on request.

## Supplementary Materials

The following supporting information can be downloaded at: https://www.mdpi.com/article/10.3390/gels10080503/s1, Figure S1: Morphology of gel micro-spheres under different concentrations of sodium alginate: (1) 0.5%, (2) 1%, (3) 1.5%, (4) 2%, and (5) 2.5%.; Figure S2: Effects of different sodium alginate concentrations on spherical adsorbent; Figure S3: Effects of different CaCl2 concentrations on spherical adsorbent; Figure S4: (a) Linear diagram for solving thermodynamic parameter Kd; (b) Linear diagram for thermodynamic parameter.
