# Supplementary material for: Preparation and Characterization of Chitosan-Modified Bentonite Hydrogels and Application for Tetracycline Adsorption from Aqueous Solution
Source: Gels. 2024 Jul 28;10(8):503. doi: 10.3390/gels10080503 (PMC11353548; doi:10.3390/gels10080503)
Supplement: Supplementary file 1 [file gels-10-00503-s001.zip › gels-3106872-supplementary.pdf]

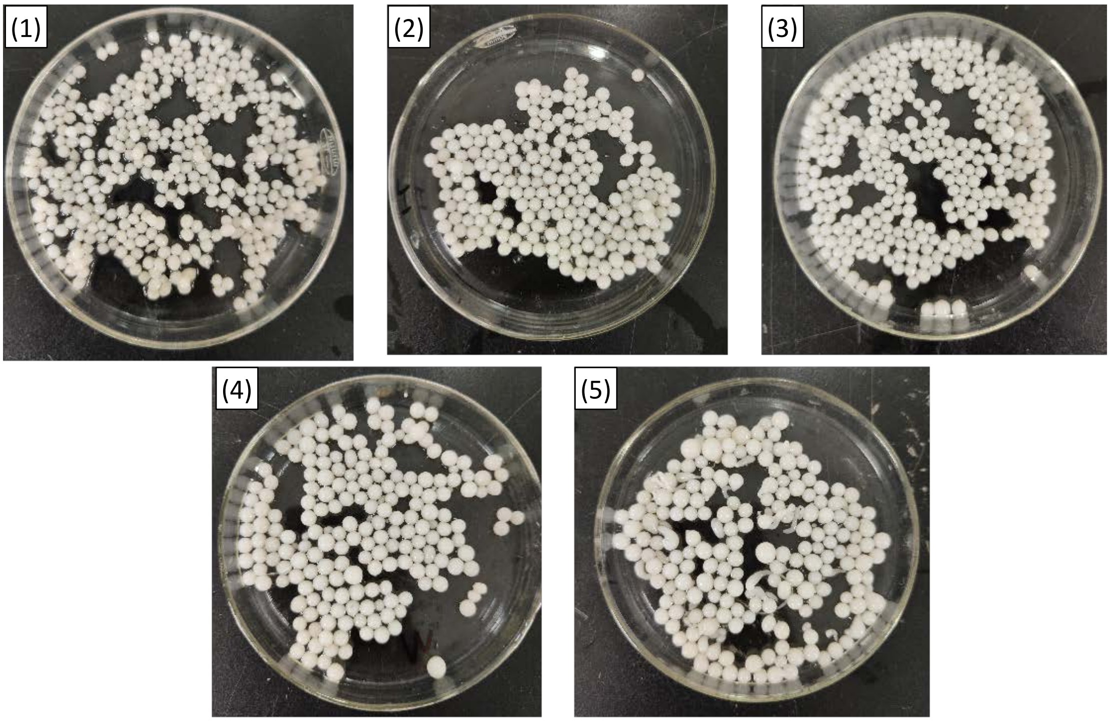

Figure S1 Morphology of gel micro-spheres under different concentrations of sodium alginate: (1) 0.5%, (2) 1%, (3) 1.5%, (4) 2%, and (5) 2.5%.

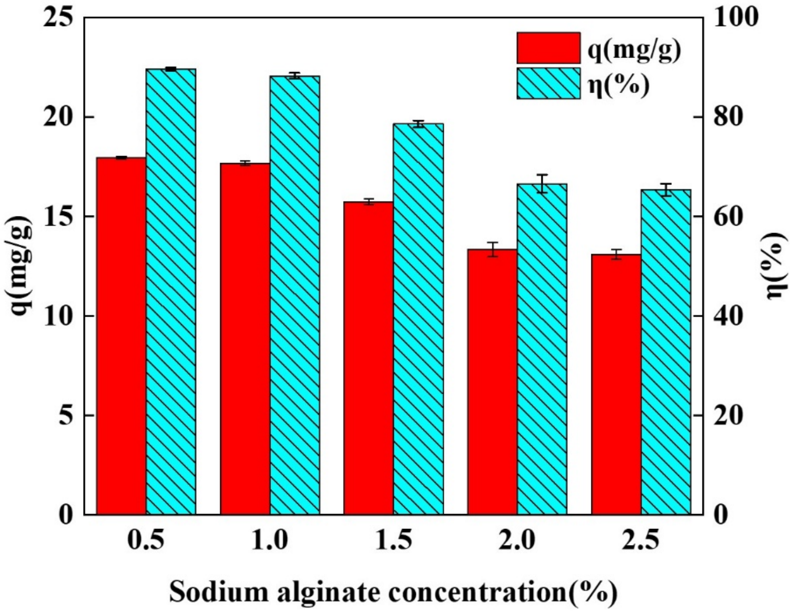

Figure S2 Effects of different sodium alginate concentrations on spherical adsorbent

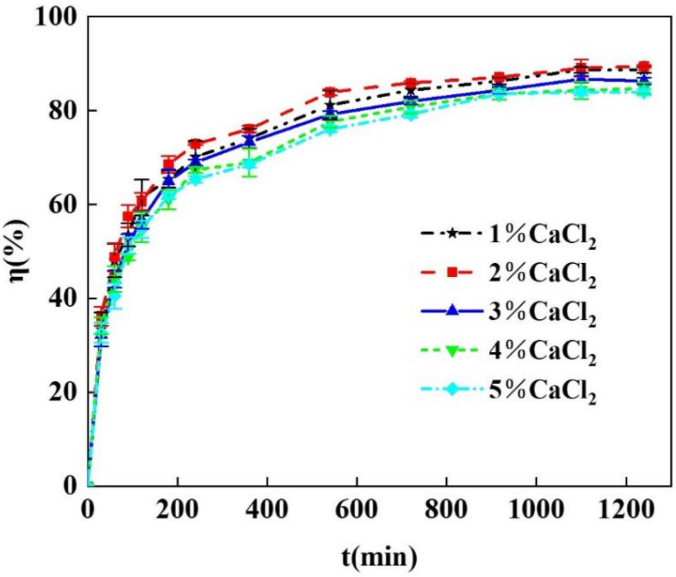

Figure S3 Effects of different $CaCl_2$ concentrations on spherical adsorbent

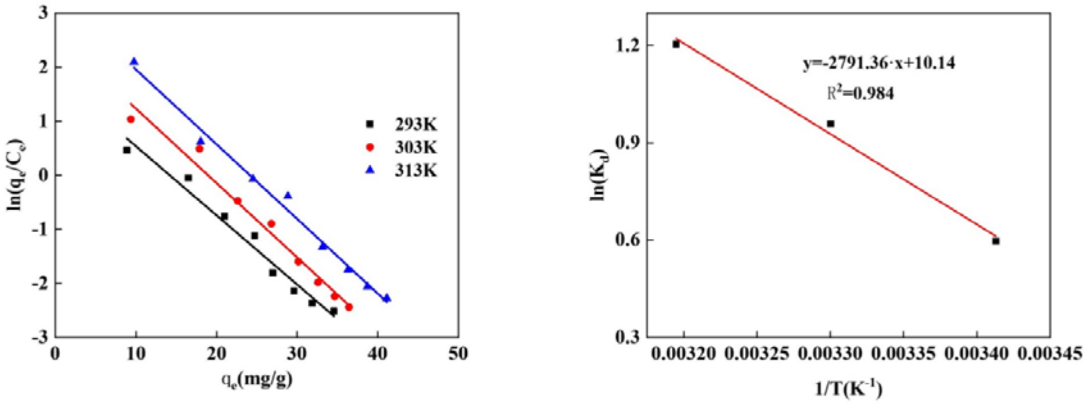

(a) (b)

Figure S4 (a) Linear diagram for solving thermodynamic parameter K_d; (b) Linear diagram for thermodynamic parameter
